# Secretory IgA and T cells targeting SARS-CoV-2 spike protein are transferred to the breastmilk upon mRNA vaccination

**DOI:** 10.1016/j.xcrm.2021.100468

**Published:** 2021-12-02

**Authors:** Juliana Gonçalves, A. Margarida Juliano, Nádia Charepe, Marta Alenquer, Diogo Athayde, Filipe Ferreira, Margarida Archer, Maria João Amorim, Fátima Serrano, Helena Soares

**Affiliations:** 1 Human Immunobiology and Pathogenesis Group, CEDOC, NOVA Medical School | Faculdade de Ciências Médicas, NOVA University of Lisbon, Lisbon, Portugal; 2 iNOVA4Health, Lisbon, Portugal; 3 Centro Hospitalar Universitário Lisboa Central, Lisbon, Portugal; 4 CHRC, CEDOC, NOVA Medical School | Faculdade de Ciências Médicas, NOVA University of Lisbon, Lisbon, Portugal; 5 Cell Biology of Viral Infection Lab, Instituto Gulbenkian de Ciência, Oeiras, Portugal; 6 Membrane Protein Crystallography Laboratory, Instituto de Tecnologia Química e Biológica, ITQB-NOVA, Oeiras, Portugal

**Keywords:** mRNA vaccine, milk-transferred SARS-CoV-2 protection, spike SIgA, milk-transferred spike-reactive T cells, memory B cells, plasmablasts, COVID-19, lactating women, maternal vaccination, breastmilk T cells

## Abstract

In view of the scarcity of data to guide decision making, we evaluated how BNT162b2 and mRNA-1273 vaccines affect the immune response in lactating women and the protective profile of breastmilk. Compared with controls, lactating women had a higher frequency of circulating RBD memory B cells and higher anti-RBD antibody titers but similar neutralizing capacity. We show that upon vaccination, immune transfer to breastmilk occurs through a combination of anti-spike secretory IgA (SIgA) antibodies and spike-reactive T cells. Although we found that the concentration of anti-spike IgA in breastmilk might not be sufficient to directly neutralize SARS-CoV-2, our data suggest that cumulative transfer of IgA might provide the infant with effective neutralization capacity. Our findings put forward the possibility that breastmilk might convey both immediate (through anti-spike SIgA) and long-lived (via spike-reactive T cells) immune protection to the infant. Further studies are needed to address this possibility and to determine the functional profile of spike T cells.

## Highlights

• Immune transfer to breastmilk occurs through spike SIgA, IgG, and T cells
• mRNA vaccines induce antibody production by the mammary mucosa
• Repeated feedings possibly lead to cumulative transfer of neutralizing spike IgA
• Vaccination induces higher frequency of RBD^+^ memory B cells in lactating women

## Graphical abstract

**Figure:**
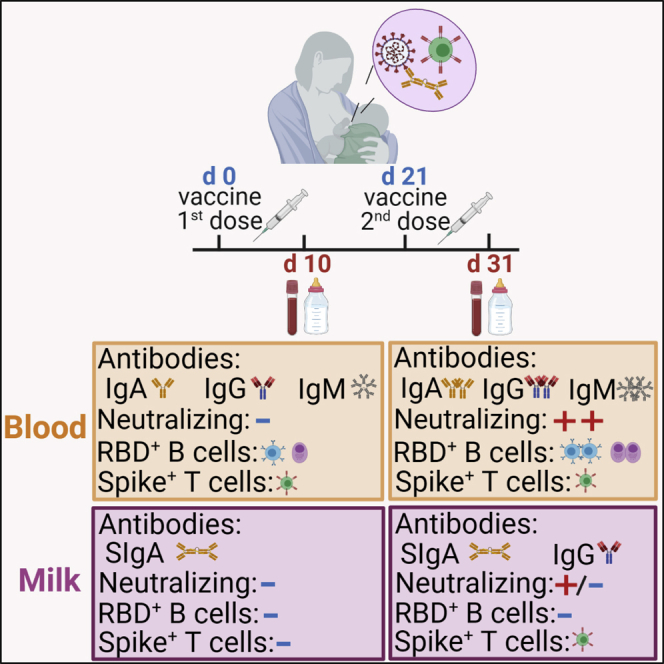

## Teaser

Gonçalves et al. show that lactating women vaccinated with the Pfizer and Moderna anti-COVID-19 mRNA vaccines transfer to breastmilk spike-reactive secretory antibodies (SIgA) and T cells. These findings advance the possibility that breastmilk might convey both immediate antibody-mediated and long-lived cellular-mediated immune protection to the infant.

## Introduction

Clinical trials of coronavirus disease 2019 (COVID-19) mRNA vaccines excluded lactating women, causing a dearth of data to guide vaccine decision making by health authorities.^1^ This is especially worrisome because infants are the group of children most affected by COVID-19.^2^^,^^3^ In view of the physiological alterations observed in lactating women and of the crucial role of breastmilk in providing immunity to the suckling infant, there is a pressing need to determine how mRNA vaccines affect immune responses in lactating mothers and to uncover the effector profile of breastmilk-transferred immune protection.

Infants have an immature immune system and rely on the transfer of maternal immune cells and antibodies via breastmilk to provide them with immunity.^4^, ^5^, ^6^, ^7^, ^8^ Human breastmilk contains a wide variety of immunoglobulins, including IgA (∼90%), IgM (∼8%), and IgG (∼2%).^9^ Although milk IgG originates mostly from blood, IgA and IgM originate predominantly from mucosa-associated lymphatic tissue (MALT) within the mammary gland.^10^^,^^11^ At mucosal sites, IgA and IgM are secreted in the form of polymeric antibodies complexed to j-chain and secretory component proteins.^10^ Whether mucosal immunity is elicited or not by vaccination depends to a great extent on the vaccination route, with intramuscular inoculation favoring a systemic immune response and intranasal or oral vaccination inducing mucosal immunity.^12^^,^^13^ Nonetheless, transfer of protective neutralizing IgA antibodies via breastmilk can occur following intramuscular vaccination against influenza.^14^ It remains to be addressed whether COVID-19 mRNA vaccine-elicited milk IgA^15^, ^16^, ^17^, ^18^, ^19^ is indeed produced by mammary MALT as secretory IgA (SIgA) or if it is provided as monomeric IgA by the blood. Moreover, it is currently unclear if vaccine-elicited milk IgA can confer immune protection to the infant through viral neutralization.

In addition to antibodies, breastmilk contains effector T cells and class-switched IgD^−^ memory B cells and plasma cells.^20^, ^21^, ^22^, ^23^, ^24^ Several lines of evidence support that milk B and T cells are capable of withstanding the gastric environment,^25^, ^26^, ^27^ enter the blood circulation,^28^^,^^29^ and are distributed into infant tissues.^30^^,^^31^ Recent studies have indicated that transfer of maternal lymphocytes via breastmilk greatly assists the newborn’s immune system.^11^ mRNA vaccines induce spike-reactive B and T cells in the blood,^32^^,^^33^ including in breastfeeding women.^34^ Regardless, whether mRNA vaccines can elicit mammary MALT T and B cell responses that could be transferred to the infant via breastmilk remains unknown.

Here we sought to gauge the effects of mRNA vaccines on the immune response of lactating women and to uncover breastmilk effector immune composition.

## Results

### COVID-19 mRNA vaccines induce production of SIgA by mammary mucosa early upon first dose administration

We collected 23 paired samples of breastmilk, pre-vaccination and after first and second mRNA vaccine administration. Demographic data are contained in Tables S1 and S2.

**Figure 1:**
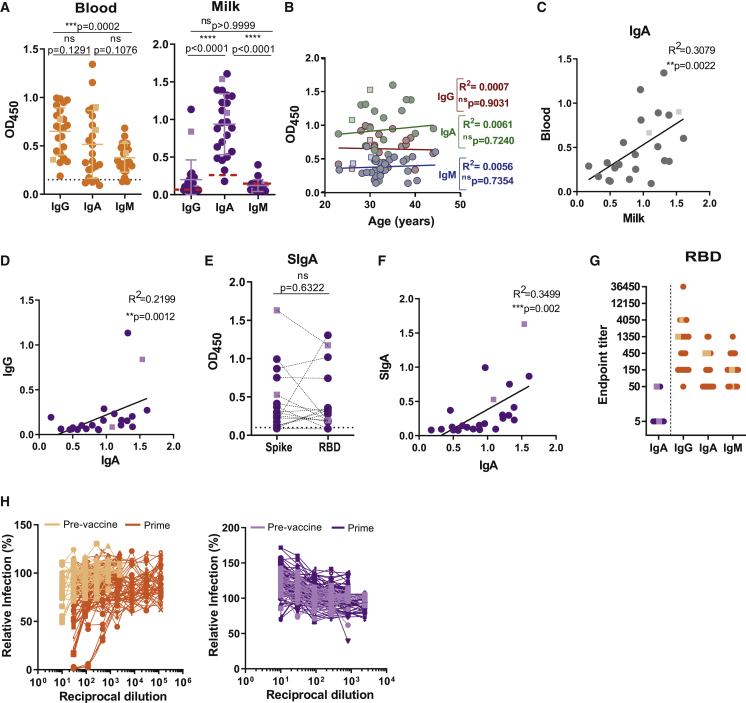
mRNA vaccines induce production of SIgA into the breastmilk early upon first dose administration (A) Anti-spike IgG, IgA, and IgM in plasma and skim milk of breastfeeding women (n = 23), measured by absorbance at 450 nm (OD_450_). (B) Correlation between anti-spike IgG, IgA, and IgM and donor’s age in years. (C) Correlation between anti-spike IgA in plasma and skim milk. (D) Correlation between anti-spike IgA and IgG in skim milk. (E) Donor-matched analysis between anti-spike and anti-RBD SIgA in skim milk. (F) Correlation between anti-spike IgA and SIgA in skim milk. (G) Endpoint titers for anti-RBD antibodies in skim milk (IgA) and plasma (IgG, IgA, and IgM). (H) Neutralization curves for plasma and skim milk. Orange, plasma; purple, skim milk. Circles, Pfizer; squares, Moderna. Dashed line, assay cutoff. n = 23 nursing women and n = 22 controls. p values determined using ANOVA, post hoc Turkey and Friedman tests, or post hoc Dunn test when comparing three groups and using parametric paired t test and non-parametric paired Wilcoxon test as appropriate. Pearson and Spearman correlations. ∗∗p < 0.01, ∗∗∗p < 0.001, and ∗∗∗∗p < 0.0001; ns, not significant.

We looked at humoral response in breastmilk and blood ∼10 days after first vaccine dose, when protection conferred by mRNA vaccines is starting.^35^^,^^36^ All lactating women had anti-spike antibodies in circulation, with 17 of 23 IgA^+^IgG^+^IgM^+^, 2 of 23 IgG^+^IgM^+^, and 1 of 23 IgA^+^IgG^+^ (Figure 1A). Similarly, 22 of 23 lactating women presented anti-spike antibodies in breastmilk, with 2 of 23 IgA^+^IgG^+^IgM^+^, 3 of 23 IgA^+^, and 17 of 23 IgA^+^IgG^+^ (Figure 1A). Anti-spike IgG, IgA, and IgM antibody levels did not correlate with donors’ age (Figure 1B). We detected a trend between milk and blood anti-spike IgA levels and within milk samples a correlation between anti-spike IgA and IgG (Figures 1C and 1D). As mRNA vaccines are better suited to inducing systemic monomeric IgA than polymeric mucosal SIgA, we sought to identify the source of IgA in the breastmilk through detection of SIgA reactive against spike and its RBD domain. Anti-spike SIgA was present in 70% of milk samples and correlated with anti-spike IgA in milk (Figures 1E and 1F).

To gain insight into the possible neutralizing properties of milk and blood antibodies, we first assessed RBD endpoint titers.^37^ Milk anti-RBD IgA endpoint titer was lower compared with the corresponding anti-RBD endpoint titers for IgA, IgG, and IgM in blood (Figure 1G). None of milk samples were neutralizing, and even though several blood samples possessed moderate (1:450) and high (>1:1,350) antibody endpoint titers, only three blood samples were neutralizing (Figure 1H).

Altogether our data show that SIgA is produced early (day 10) by the mammary mucosa in response to the first dose of mRNA vaccine and indicate that mRNA vaccines are capable of inducing a local immune response by the mammary MALT.

### Unconcentrated neutralizing IgA can be found in milk after second vaccine dose

**Figure 2:**
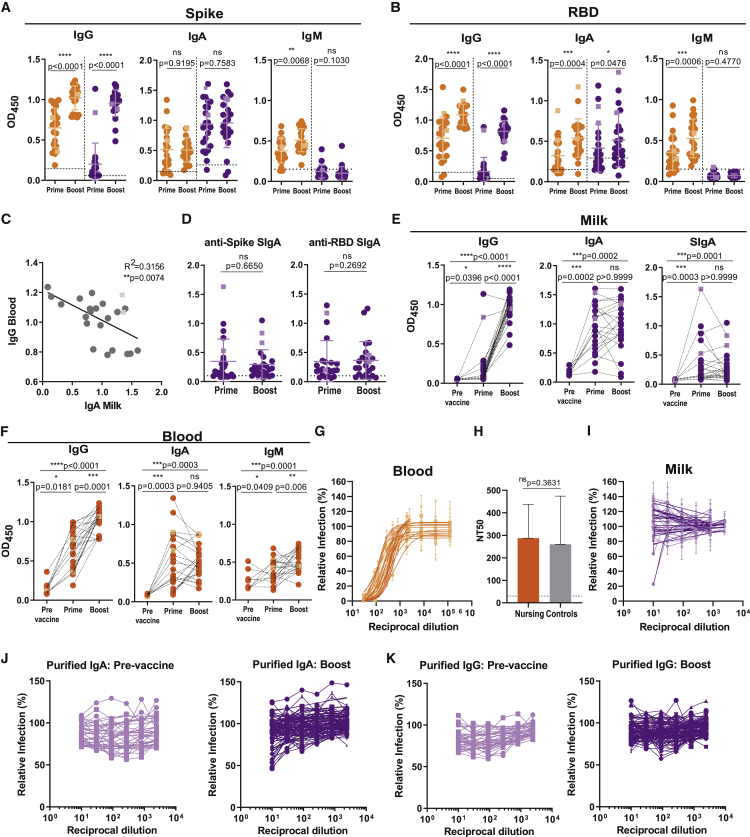
Neutralizing antibodies are found in blood and less frequently in milk after second vaccine dose (A) Anti-spike IgG, IgA, and IgM at ∼10 days after first (prime) and second (boost) vaccine doses in plasma and in skim milk, measured by absorbance at 450 nm (OD_450_). (B) Anti-RBD IgG, IgA, and IgM performed as in (A). (C) Correlation between anti-spike IgA in skim milk versus anti-spike IgG in the blood. (D) Comparison between anti-spike- and anti-RBD SIgA at ∼10 days after first (prime) and second (boost) vaccine doses, measured as in (A). (E) Donor-matched analysis of anti-spike IgG, IgA, and SIgA, pre-vaccination and after first (prime) and second (boost) vaccine doses, in skim milk. (F) Donor-matched analysis of anti-spike IgG, IgA, and IgM, pre-vaccination and after first (prime) and second (boost) vaccine doses, in blood. (G) Plasma neutralization curves. (H) Plasma neutralization titers (NT_50_) in nursing and control women. (I) Skim milk neutralization curves. (J) Neutralization curves for skim milk purified IgA concentrated 5-fold, pre-vaccination and ∼10 days after vaccine boost. (K) Neutralization curves for skim milk purified IgG concentrated 5-fold, pre-vaccination and ∼10 days after vaccine boost. Orange, plasma; purple, skim milk. Circles, Pfizer; squares, Moderna. Dashed line, assay cutoff. n = 23 nursing women and n = 22 controls. p values determined using parametric paired t test and non-parametric paired Wilcoxon test as appropriate and using ANOVA, post hoc Holm-Sidak and Kruskal-Wallis tests, and post hoc Dunn test when comparing three groups. Spearman correlation. ∗p < 0.05, ∗∗p < 0.01, ∗∗∗p < 0.001, and ∗∗∗∗p < 0.0001; ns, not significant.

Although circulating neutralizing IgG to mRNA vaccine is optimally detected after boost,^38^ it is currently unclear whether vaccine-induced milk antibodies are neutralizing.^15^^,^^16^^,^^19^ Contemporaneous with the IgG surge in the blood at ∼10 days after vaccine boost, we detected anti-spike and anti-RBD IgG in milk (Figures 2A and 2B). In contrast, the frequency and levels of spike-reactive IgA remained constant in blood and milk, even though we observed a slight increase in milk anti-RBD IgA (Figures 2A–2E). Similarly, milk SIgA levels recognizing spike and its RBD domain remained constant upon vaccine boost (Figure 2D). Interestingly, anti-spike IgA in breastmilk inversely correlated with circulating anti-spike IgG (Figure 2C). This might be because higher induction of circulating IgG might prevent spike protein from reaching the mammary MALT and effectively induce local antibody production. Donor paired analysis shows that although milk anti-spike IgG was boosted, milk anti-spike IgA and SIgA remained constant following second vaccine dose (Figure 2E). Nonetheless the frequency of milk samples with spike SIgA increased from 70% to 87%. Vaccination induced a similar antibody profile in blood, with vaccine boost increasing anti-spike IgG but not affecting IgA (Figure 2F).^39^ IgM was poorly induced upon vaccination (Figure 2F).

As expected, all plasma from fully vaccinated lactating women was neutralizing (half-maximal neutralization titer [NT_50_] = 238.69, interquartile range [IQR] 148.47–383.36) and comparable with an age-matched female cohort (NT_50_ = 215.39, IQR 135.34–303.51) (Figures 2G and 2H) and previous reports for mRNA vaccines.^33^^,^^38^^,^^40^ Importantly, only one milk sample displayed weak neutralizing activity (Figure 2I). To identify which milk Ig could potentially neutralize SARS-CoV-2, we purified IgA and IgG by affinity chromatography and concentrated 5-fold prior to running neutralization assays. Weak neutralization could be detected in only three IgA fractions, which clustered around the highest SIgA levels (Figures 2J and 2K; Table S3). Next, we purified milk IgA using size exclusion chromatography (SEC) (Figures S1A and S1B). Purified IgA from pre- and post-vaccination samples eluted in a single peak corresponding to polymeric SIgA, suggesting that vaccination did not result in an influx of monomeric IgA from the blood (Figures S1C and S1D).

Our data indicate that anti-spike SIgA titers were unaffected by vaccine boosting. Although the concentration of spike-reactive IgA in breastmilk might not be sufficient to directly neutralize viral infection, our data suggest that cumulative transfer of IgA through feeding might provide the infant with effective SARS-CoV-2 neutralization.

### Lactating women have higher frequency of circulating RBD-reactive memory B cells and anti-RBD antibodies

**Figure 3:**
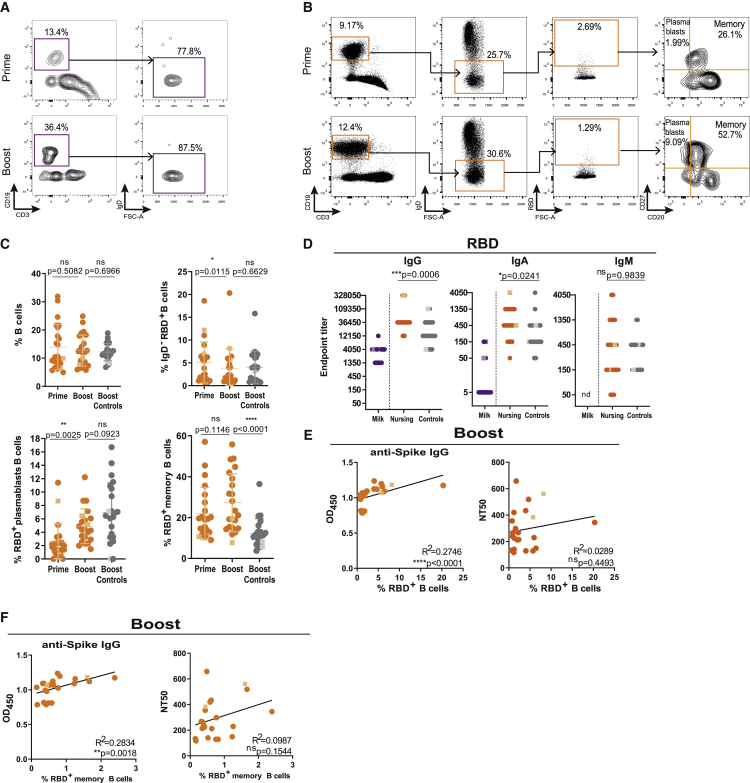
Lactating women have higher frequencies of RBD-reactive memory B cells and anti-RBD antibodies in circulation (A) Gating strategy for CD3^−^CD19^+^IgD^−^ B cells in skim milk after first (prime) and second (boost) vaccine doses. (B) Gating strategy for circulating RBD-reactive CD3^−^CD19^+^IgD^−^CD20^−^CD27^+^ plasmablasts and CD3^−^CD19^+^IgD^−^CD20^+^CD27^+^ memory B cells. (C) Cumulative frequency of circulating total B cells (top left), RBD-reactive B cells (top right), plasmablasts (bottom left), and memory B cells (bottom right) after first (prime) and second (boost) vaccine doses for nursing and after the second (boost) vaccine dose for controls. (D) Endpoint titers for anti-RBD IgG, IgA, and IgM in skim milk and plasma of nursing women and in plasma of controls. nd, non-detectable. (E) Correlation between anti-spike IgG and neutralization titers with the frequency of RBD-reactive B cells upon vaccine boosting. (F) Correlation between anti-spike IgG and neutralization titers (NT_50_) and the frequency of RBD-reactive memory B cells upon vaccine boosting. Circles, Pfizer vaccine; squares, Moderna vaccine. n = 23 nursing women and n = 22 controls. p values determined using non-parametric paired Wilcoxon test, t test, and Man-Whitney test as appropriate. Pearson and Spearman correlations. ∗p < 0.05, ∗∗p < 0.01, ∗∗∗p < 0.001, and ∗∗∗∗p < 0.0001; ns, not significant.

Although human IgA secreting B cells are preferentially retained in the mammary gland, functional IgG secreting B cells can be found in higher frequencies in breastmilk.^11^^,^^22^^,^^41^ We sought to evaluate the presence of RBD-reactive B cells in the milk following vaccination. We could detect B cells in only 5 of 23 milk samples, which were overwhelmingly IgD^22^ (Figure 3A). Because of the limited number of B cells detected, we were not able to assess the presence of RBD-reactive milk B cells.

Mainly because of hormonal changes, lactating women display distinct immune responses.^42^ In the blood, a clear population of RBD-binding IgD^−^ B cells could be detected after first vaccine dose, which remained unaltered upon boost (Figures 3B and 3C). Both RBD-reactive plasmablasts and memory B cells were detectable after first vaccine dose, with only plasmablasts increasing in frequency upon boost (Figures 3B and 3C). Compared with controls, breastfeeding women have a higher frequency of memory B cells and higher titers of circulating anti-RBD IgA and IgG (Figures 3C and 3D). Moreover, RBD-reactive memory B cells, and overall RBD-reactive B cells, correlated with anti-spike IgG levels (Figures 3E and 3F) but not with neutralization titers (Figures 3E and 3F).

Altogether, lactating women had higher frequencies of RBD-reactive circulating memory B cells and higher RBD-IgG antibodies compared with controls.

### Spike-specific T cells are transferred through breastmilk

**Figure 4:**
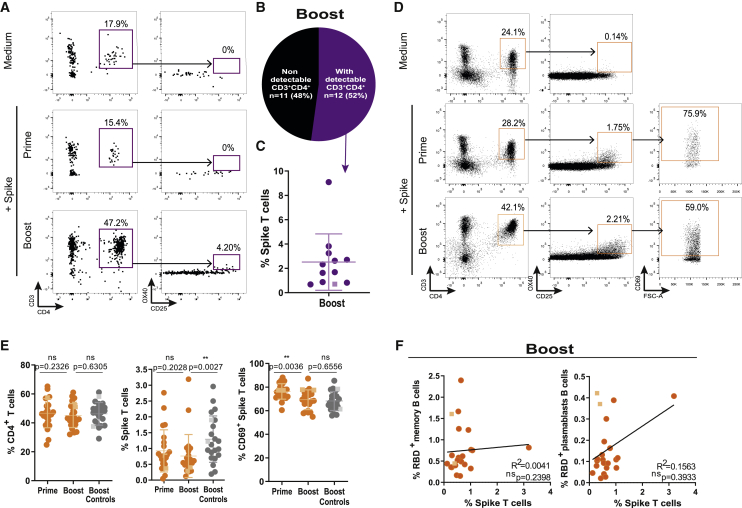
Spike-specific T cells are transferred to breastmilk after vaccine boost (A) Gating strategy for CD3^+^CD4^+^OX40^+^CD25^+^ spike-specific T cells in skim milk after first (prime) and second (boost) vaccine doses. (B) Frequency of milk samples with detectable versus non-detectable CD3^+^CD4^+^ T cells. (C) Cumulative frequency of spike-specific T cells in skim milk after vaccine boost. (D) Gating strategy of circulating for spike-specific T cells, after first (prime) and second (boost) vaccine doses. (E) Cumulative frequencies of T cells (left), spike-specific T cells (middle), and CD69^+^ spike-specific T cells (right) after first (prime) and second (boost) vaccine doses in nursing women and post-boost in controls. (F) Correlation between the frequency of RBD-reactive memory B cells (right) and plasmablasts (left) and the frequency of spike-specific T cells after boost. Circles, Pfizer vaccine; squares, Moderna vaccine. n = 23 nursing women and n = 22 controls. p values determined using parametric paired and unpaired t tests, non-parametric paired Wilcoxon test, and Mann-Whitney test as appropriate. Spearman correlation. ∗∗p < 0.01; ns, not significant.

Emerging evidence suggests the requirement of both antibody-mediated and T cell-mediated immunity for effective protection against SARS-CoV-2.^43^^,^^44^ To detect spike-specific CD4^+^ T cells, we used an activation induced marker (AIM) assay using OX40 and CD25 dual expression to detect spike reactivity.^45^, ^46^, ^47^ We could robustly detect CD4^+^ T cells in the milk of only 12 donors (52%) (Figures 4A and 4B). The absence of T cell detection in the other 11 samples was likely due to insufficient milk volume available. After second vaccine dose, spike-reactive T cells could be identified in all milk samples with detectable T cells, with frequencies ranging from 0.7% to 9.1% (Figures 4A and 4C), indicating that spike T cells are transferred to breastmilk upon mRNA vaccination.

All lactating women possessed spike T cells (median 0.76%, IQR 0.5%–1.19%) in circulation after first vaccine dose, and their frequency was not altered by subsequent boost (Figures 4D and 4E), even though their activation state, measured by CD69 expression, was decreased (Figures 4D and 4E). Curiously, it appears that after vaccine boost, breastfeeding women have fewer spike-reactive CD4^+^ T cells compared with vaccinated controls (Figures 4D and 4E). In view of the role of CD4^+^ T cells in B cell effector differentiation, we looked for an association between circulating spike-reactive T cells and RBD-reactive B cells. There was no correlation between spike T cells and RBD-plasmablasts or memory B cells (Figure 4F).

Altogether our data show that in addition to antibodies, milk also contains spike-reactive T cells. These spike-reactive T cells might transfer long-lived immunity to the suckling infant.

## Discussion

Within the pediatric population, infants present the highest COVID-19 fatality rate.^2^^,^^3^^,^^48^ Yet lactating women were initially advised to discontinue breastfeeding following COVID-19 mRNA vaccination.^49^ How mRNA vaccines affect lactating women, and the breadth and effector profile of the milk-transferred immune response, remains poorly understood. Through a combination of serology, virus neutralization, flow cytometry, and SEC we identified the secretory and neutralizing properties of breastmilk-transferred antibodies and cellular immunity induced by mRNA vaccination.

The function of mammary MALT is to provide protective immunity to the infant through the secretion of polymeric antibodies complexed to j-chain and secretory component proteins.^10^ The secretory component is essential to ensure that milk antibodies survive the gastric environment and are effectively transferred to the infant.^10^ Spike-reactive SIgA was detected in breastmilk of COVID-19 patients,^50^ but whether SIgA was similarly present upon mRNA vaccination had remained unaddressed. We detected spike-reactive SIgA in 87% of milk samples, reinforcing the suggestion that mRNA vaccines can elicit immune responses by mammary and oral^51^ mucosa. We then explored the ability of milk antibodies to neutralize SARS-CoV-2. Even though only 1 of 23 milk samples was directly neutralizing, purification and concentration of IgA increased its neutralization capacity. Suggesting that cumulative transfer of IgA through breastmilk might provide the infant with effective neutralization capacity. Compared with COVID-19 infection,^34^^,^^52^ milk neutralizing capabilities post-vaccination appear to be weaker. This is consistent with the fact that spike-reactive SIgA does not increase upon vaccine boost, indicating a T cell-independent production. Under these conditions, mucosal-produced SIgA has been described as polyreactive.^10^^,^^53^ As SARS-CoV-2 infection effectively primes airway and gut mucosa immunity, the two sources of mammary T and B cells,^11^ it is likely that milk SIgA production is primed in a T cell-dependent manner and thus presents higher neutralization capacity.^54^^,^^55^

An important and until now completely unexplored route for milk-transferred COVID-19 immunity is through T cells. Milk-transferred lymphocytes can survive the adverse environment of the digestive tract and seed in the infant’s tissues.^27^, ^28^, ^29^, ^30^, ^31^, ^32^, ^33^ This is due to a combination of a biochemical reaction between the infant’s saliva and breastmilk that protects lymphocytes from acid injury,^4^, ^5^, ^6^ a decrease in enzyme and acid content in the infant’s digestive tract,^56^^,^^57^ and an increase in gut permeability.^28^ CD4^+^ T cells are crucial in mediating mRNA vaccine protection,^40^^,^^58^^,^^59^ especially in the setting of suboptimal neutralizing antibodies.^60^ It is possible that spike-reactive T cells that have been transferred to breastmilk might mediate protection from infection by seeding in the infant’s upper respiratory tract and gut.

Lactating women display a unique immune state, evolved to provide immunity to the infant. We detected higher RBD-reactive IgG and IgA titers in lactating women, but their neutralization titers were undistinguishable from those of controls, which is consistent with previous findings.^19^ In addition, we found that lactating women had higher frequencies of RBD-reactive memory B cells in circulation, which correlated with their anti-spike IgG titers. Even though the reasons for increased frequencies of RBD-reactive memory B cells are likely multifactorial, increased levels of milk-inducing prolactin have been associated with increased antibody titers and activated “memory”-like B and T cells.^61^, ^62^, ^63^, ^64^, ^65^, ^66^ As memory B cells have been proposed to play a key role in mounting recall responses to COVID-19 mRNA vaccines,^38^ further studies will be needed to determine if these cellular differences are maintained in the medium term and whether they will affect long-term protection.

Here we show that upon mRNA vaccination, immune transfer to breastmilk occurs through a combination of spike-reactive SIgA and T cells. Together, these two lines of defense might synergize in conferring both immediate (SIgA) and long-lived (T cells) protection to the nursing infant. Our data support the view that lactating women’s immune response to the vaccine is skewed toward long-lasting memory B cell response. The clinical translatability of our findings is multifold. First, our data provide compelling evidence that COVID-19 mRNA vaccine induces a stronger memory B cell response in lactating women, hinting that lactating women might sustain effective B cell responses longer, which might affect boost vaccination scheduling. Second, this work suggests that protection transferred to milk occurs in the form of antibodies and T cells. As memory T cells are long lived, this opens the possibility that milk-transferred protection might still be present in the infant even after weaning. Third, our results suggest that repeated feedings might confer neutralizing protection mediated by SIgA. This has important clinical implications, as SIgA is more resistant to gastric degradation than IgG, meaning that neutralizing protection might be transferred through breastmilk even after the introduction of solid foods.

### Limitations of the study

Our results represent only a snapshot of what is likely a dynamic immune response. However, we show that transfer of SIgA and T cells to milk spans a wide breastfeeding window. Because of inequal representativity, we were unable to explore any differences in milk-transferred protection between the two mRNA vaccines. Future research using larger sample sizes and longitudinal designs is needed to determine whether spike-reactive SIgA and T cells contained in breastmilk confer immunity to the infant and to elucidate their effector mechanisms.

## STAR★Methods

### Key resources table

**Table:** 

REAGENT or RESOURCE	SOURCE	IDENTIFIER
**Antibodies**		

Mouse monoclonal purified anti-human CD28 (Clone CD28.2)	BioLegend	Cat#302933; RRID: AB_11150591
Mouse monoclonal anti-human CD3 (Clone UCHT1)	BioLegend	Cat#300424; RRID: AB_493741
Mouse monoclonal anti-human CD4 (Clone SK3)	BioLegend	Cat#344665; RRID: AB_2876651
Mouse monoclonal anti-human CD19 (Clone SJ25C1)	BioLegend	Cat#363026; RRID: AB_2564255
Mouse monoclonal anti-human CD20 (Clone 2H7)	BioLegend	Cat#302342; RRID: AB_2562602
Mouse monoclonal anti-human CD25 (Clone M-A251)	BioLegend	Cat#356108; RRID: AB_2561975
Mouse monoclonal anti-human CD27 (Clone O323)	BioLegend	Cat#302810; RRID: AB_314302
Mouse monoclonal anti-human CD69 (Clone FN50)	BioLegend	Cat#310930; RRID: AB_2561909
Mouse monoclonal anti-human CD134 (Clone Ber-ACT35)	BioLegend	Cat#350028; RRID: AB_2629633
Mouse monoclonal anti-human IgD (clone IA6-2)	BioLegend	Cat#348249; RRID: AB_2876661
Goat polyclonal ACE-2	R&D Systems	Cat#AF933; RRID: AB_355722
Goat Anti-Human IgG	Abcam	Cat#ab97225; RRID: AB_10680850
Goat Anti-Human IgA	Abcam	Cat#ab97215; RRID: AB_10680847
Goat Anti-Human IgM	Abcam	Cat#ab97205; RRID: AB_10695942
Anti-IgA Secretory Component	Abcam	Cat#ab3924; RRID: AB_2261963
Anti-mouse IgG1 (clone RMG1-1)	BioLegend	Cat#406601; RRID: AB_315060
Goat Anti-Mouse IgG HRP	Bio Rad	Cat#1706516; RRID: AB_11125547
Donkey anti-Goat IgG	Invitrogen	Cat#A-11057; RRID: AB_142581
Fixable Viability Dye eFluor 506	Invitrogen	Cat#65-0866-18; RRID: N/A

**Chemicals, peptides, and recombinant proteins**		

Peptide M / Agarose	InvivoGen	Cat#gel-pdm-2; RRID: N/A
Protein G Agarose	Thermo Scientific	Cat#20398; RRID: N/A
SARS-CoV-2 Spike CS + PP	iBET Bioproduction Unit	N/A
SARS-CoV-2 RBD	iBET Bioproduction Unit	N/A

**Critical commercial assays**		

Alexa Fluor 488 Antibody Labeling Kit	Invitrogen	Cat#A20181; RRID: N/A

**Experimental models: Cell lines**		

Human Embryonic Kidney 293T	Prof Paul Digard, Roslin Institute, UK	N/A
Human Embryonic Kidney 293ET	Dr Colin Adrain, Instituto Gulbenkian de Ciência, Portugal	N/A

**Recombinant DNA**		

Plasmid pLEX-ACE2	Dr. Maria João Amorim, Instituto Gulbenkian de Ciência, Portugal; Alenquer et al., 2021	N/A
Plasmid psPAX2	Dr. Luís Moita, Instituto Gulbenkian de Ciência, Portugal	N/A
Plasmid pVSV.G	Dr. Luís Moita, Instituto Gulbenkian de Ciência, Portugal	N/A

**Software and algorithms**		

FlowJo V10.7.3	BD Biosciences	https://www.flowjo.com/solutions/flowjo; RRID: SCR_008520
Prism V9.00	GraphPad	http://www.graphpad.com/; RRID: SCR_002798

### Resource availability

#### Lead contact

Further information and requests for resources and reagents should be directed to and will be fulfilled by the lead contact (helena.soares@nms.unl.pt).

#### Materials availability

This study did not generate new unique reagents.

### Experimental model and subject details

We collected 23 paired samples of breastmilk and blood on two separate occasions. Coinciding with the priority vaccination of health care workers, we collected 14 paired samples between December 2020 and February 2021, a median of 10 days after first (interquartile range (IQR), 8-12 days) and second (IQR, 9-10 days) mRNA vaccine administration, as previously described^67^ (Table S1). The second collection interval occurred upon opening of the vaccination to the general population from June to September 2021 and participants were recruited through social media platforms, pre-natal support groups and/or word of mouth. In this second period, we collected 9 paired samples of breastmilk and blood, ∼11 days after the first (IQR, 10-15 days) and ∼15 days after the second (IQR, 11-25 days) mRNA vaccine administration (Table S2). Of the 22 control participants, 20 received with Pfizer BTN162b2 and 2 the Moderna mRNA-1273 vaccine. Blood was collected by venopuncture in EDTA tubes and breastmilk was expressed with breast pump into sterile containers. Biospecimens were immediately processed. All participants provided informed consent and all procedures were approved by NOVA Medical School ethics committee (11/2021/CEFCM and 112/2021/CEFCM), in accordance with the provisions of the Declaration of Helsinki and the Good Clinical Practice guidelines of the International Conference on Harmonization.

### Method details

#### Blood and breastmilk cell isolation

Peripheral blood mononuclear cells (PBMCs) were isolated by density gradient centrifugation (Biocoll, Merck Millipore).^68^ Breastmilk cells were isolated by centrifugation. Plasma and skim milk samples were stored at −80°C or −20°C, respectively, until further analysis. PBMCs and breastmilk mononuclear cells were suspended in freezing media (10% DMSO in FBS) and stored at −80°C until subsequent analysis.

#### Flow cytometry

RBD was labeled with an available commercial kit according to manufactor’s instructions (Life technologies, A20181). For detection of SARS-CoV-2 reactive T cells, cryopreserved PBMCs were rested for 1h at 37°C and then stimulated overnight with either 1 μg/mL of spike protein plus 5 μg/mL of anti-CD28 (CD28.2, BioLegend) cross-linked with 2.5 μg/mL of anti-mouse IgG1 (RMG1-1, BioLegend) or with medium (negative control). PBMCs were stained with a fixable viability dye eFluor™ 506 (Invitrogen) and surface labeled with the following antibodies all from BioLegend: anti-CD3 (UCHT1), anti-CD4 (SK3), anti-OX40 (Ber-ACT35), anti-CD25 (M-A251), anti-CD69 (FN50), anti-CD19 (SJ25C1), anti-IgD (IA6-2), anti-CD27 (O323) and anti-CD20 (2H7) and also with labeled RBD as described above. Cells were washed, fixed with 1% PFA and acquired in BD FACS Aria III (BD Biosciences) and analyzed with FlowJo v10.7.3 software (Tree Star).

#### ELISA

Antibody binding to SARS-CoV-2 trimeric spike protein or its RBD domain was assessed by a previously described in-house ELISA assay^37^ based on the protocol by Stadlbauer et al.^69^ Briefly, 96-well plates (Nunc) were coated overnight at 4°C with 0.5 μg/ml of trimeric spike or RBD. After blocking with 3% BSA diluted in 0.05% PBS-T, 1:50 diluted plasma and 1:5 diluted skim milk, were added and incubated for 1 h at room temperature. Plates were washed and incubated for 30 min at room temperature with 1:25,000 dilution of HRP-conjugated anti-human IgA, IgG and IgM antibodies (Abcam, ab97225/ab97215/ab97205) or with 1:10,000 dilution of anti-human SIgA (Abcam ab3924) followed by a 30 min incubation with HRP-labeled secondary antibody (Biorad, 706516) at 1:5,000 dilution in 1% BSA 0.05% PBS-T. Plates were washed and incubated with TMB substrate (BioLegend), stopped by adding phosphoric acid (Sigma) and read at 450nm. Cut-off for plasma samples resulted from the mean of OD_450_ values from negative controls plus 3 times the standard deviation.^37^ Cut-off for skim milk samples resulted from the median of OD_450_ values from unvaccinated controls plus 2 times the standard deviation.^51^ Endpoint titers were established using a 3-fold dilution series starting at 1:50 and ending at 1:109,350 and defines as the last dilution before the signal dropped below OD_450_ of 0.15. This value was established using plasma from pre-pandemic samples collected from subjects not exposed to SARS-CoV-2.^37^ For samples that exceeded an OD_450_ of 0.15 at last dilution (1:109,350), end-point titter was determined by interpolation.^70^ As previously described,^37^ in each assay we used 6 internal calibrators from 2 high-, 2 medium- and 2 low-antibody producers that had been diagnosed for COVID-19 through RT-PCR of nasopharyngeal and/or oropharyngeal swabs. As negative controls, we used pre-pandemic plasma samples collected prior to July 2019.

#### Purification of milk IgA and IgG

IgA and IgG from breast milk samples were purified through Peptide M/Agarose (Invivogen) or Protein G (ThermoScientific), respectively, according to the manufacturer’s instructions. Briefly 1 mL of skim milk was incubated with 2 mL of Peptide M/Agarose or Protein G and incubated for 20 min. Peptide M/Agarose and Protein G beads were washed 3 times with wash buffer (10mM sodium phosphate 150mM sodium chloride; pH 7.2) and eluted in fractions of 500 μL with 0.1M glycine pH 2.76. The pH of the collected fractions was adjusted to 7 with 1M TRIS; pH 8.83, pooled IgA and IgG fractions were washed with PBS and concentrated using Amicon Ultra with a 100 kDa membrane (Millipore). All steps were carried out at 4°C. Skim milk and IgA and IgG milk fractions were analyzed on non-reducing polyacrylamide gel electrophoresis (BN-PAGE) on NativePAGE 4%–16% Bis-Tris gels (ThermoFisher Scientific) with NativeMark (ThermoFisher Scientific) as molecular weight marker and stained with ProBlue Safe Stain (Giotto Biotech).

#### Production of ACE2 expressing 293T cells

Production of 293T cells stably expressing human ACE2 receptor was done as previously described.^71^ Briefly, VSV-G pseudotyped lentiviruses encoding human ACE2, 293T cells were transfected with pVSV-G, psPAX2 and pLEX-ACE2 using jetPRIME (Polyplus), according to manufacturer’s instructions. Lentiviral particles in the supernatant were collected after 3 days and were used to transduce 293T cells. Three days after transduction, puromycin (Merck, 540411) was added to the medium, to a final concentration of 2.5 μg/ml, to select for infected cells. Puromycin selection was maintained until all cells in the control plate died and then reduced to half. The 293T-Ace2 cell line was passaged six times before use and kept in culture medium supplemented with 1.25 μg/ml puromycin.

#### Production of spike pseudotyped lentivirus

To generate spike pseudotyped lentiviral particles, 6x10^6^ 293ET cells were co-transfected with 8.89 μg pLex-GFP reporter, 6.67 μg psPAX2, and 4.44 μg pCAGGS-SARS-CoV-2-S_trunc_ D614G, using jetPRIME according to manufacturer’s instructions. The virus-containing supernatant was collected after 3 days, concentrated 10 to 20-fold using Lenti-XTM Concentrator (Takara, 631231), aliquoted and stored at −80°C. Pseudovirus stocks were titrated by serial dilution and transduction of 293T-Ace2 cells. At 24h post transduction, the percentage of GFP positive cells was determined by flow cytometry, and the number of transduction units per mL was calculated.

#### Neutralization assay

Heat-inactivated plasma, skim milk, and purified milk IgA and IgG fractions were four-fold serially diluted and then incubated with spike pseudotyped lentiviral particles for 1h at 37°C. The mix was added to a pre-seeded plate of 293T-Ace2 cells, with a final MOI of 0.2. At 48h post-transduction, the fluorescent signal was measured using the GloMax Explorer System (Promega). The relative fluorescence units were normalized to those derived from the virus control wells (cells infected in the absence of plasma or skim breast milk), after subtraction of the background in the control groups with cells only.

### Quantification and statistical analysis

Statistical analysis was performed by using GraphPad Prism v9.00. First, we tested the normality of the data by using D’Agostingo & Pearson normality test, by checking skewness and kurtosis values and visual inspection of data. Then, if the samples followed a normal distribution, we chose the appropriate parametric test; otherwise, the non-parametric counterpart was chosen. In two groups comparison: for paired data the Wilcoxon matched-pairs signed-rank test and paired t test were used; for unpaired data, Man-Whitney test and the unpaired t test were used. For multiple groups comparison, repeated-measures one-way analysis of variance (ANOVA) with posttest Turkey’s and Holm-Sidák’s multiple comparisons or Friedman or Kruskal-Wallis tests with posttest Dunn’s multiple comparisons were used as indicated. For correlations, Pearson or Spearman tests were used as described. The half-maximal neutralization titer (NT_50_), defined as the reciprocal of the dilution at which infection was decreased by 50%, was determined using four-parameter nonlinear regression (least-squares regression without weighting; constraints: bottom = 0). Spearman and Pearson correlation test were used in correlation analysis. The choice of each test was dependent on the underlying distribution and is indicated in the legend of the figures.

## Data Availability

All data reported in this paper will be shared by the lead contact upon request. This paper does not report original code. Any additional information required to reanalyze the data reported in this paper is available from the lead contact upon request.

## Supplemental information

Document S1. Figure S1 and Tables S1–S3

Document S2. Article plus supplemental information

## References

[1] Riley L.E., Jamieson D.J. (2021). Inclusion of pregnant and lactating persons in COVID-19 vaccination efforts. Ann. Intern. Med..

[2] de Siqueira Alves Lopes A., Fontes Vieira S.C., Lima Santos Porto R., Santana Santos V., Fontes Leite D.C., Eduardo Cuevas L., Queiroz Gurgel R. (2021). Coronavirus disease-19 deaths among children and adolescents in an area of Northeast, Brazil: why so many?. Trop. Med. Int. Health.

[3] Dong Y., Mo X., Hu Y., Qi X., Jiang F., Jiang Z., Tong S. (2020). Epidemiology of COVID-19 among children in China. Pediatrics.

[4] Ghosh M.K., Nguyen V., Muller H.K., Walker A.M. (2016). Maternal milk T cells drive development of transgenerational Th1 immunity in offspring thymus. J. Immunol..

[5] Cabinian A., Sinsimer D., Tang M., Zumba O., Mehta H., Toma A., Sant’Angelo D., Laouar Y., Laouar A. (2016). Transfer of maternal immune cells by breastfeeding: maternal cytotoxic T lymphocytes present in breast milk localize in the Peyer’s patches of the nursed infant. PLoS ONE.

[6] Arvola M., Gustafsson E., Svensson L., Jansson L., Holmdahl R., Heyman B., Okabe M., Mattsson R. (2000). Immunoglobulin-secreting cells of maternal origin can be detected in B cell-deficient mice. Biol. Reprod..

[7] Schlesinger J.J., Covelli H.D. (1977). Evidence for transmission of lymphocyte responses to tuberculin by breast-feeding. Lancet.

[8] Ohsaki A., Venturelli N., Buccigrosso T.M., Osganian S.K., Lee J., Blumberg R.S., Oyoshi M.K. (2018). Maternal IgG immune complexes induce food allergen-specific tolerance in offspring. J. Exp. Med..

[9] Goldsmith S.J., Dickson J.S., Barnhart H.M., Toledo R.T., Eiten-Miller R.R. (1983). IgA, IgG, IgM and lactoferrin contents of human milk during early lactation and the effect of processing and storage. J. Food Prot..

[10] Brandtzaeg P. (2007). Induction of secretory immunity and memory at mucosal surfaces. Vaccine.

[11] Laouar A. (2020). Maternal leukocytes and infant immune programming during breastfeeding. Trends Immunol..

[12] Su F., Patel G.B., Hu S., Chen W. (2016). Induction of mucosal immunity through systemic immunization: phantom or reality?. Hum. Vaccin. Immunother..

[13] Krammer F. (2020). SARS-CoV-2 vaccines in development. Nature.

[14] Schlaudecker E.P., Steinhoff M.C., Omer S.B., McNeal M.M., Roy E., Arifeen S.E., Dodd C.N., Raqib R., Breiman R.F., Zaman K. (2013). IgA and neutralizing antibodies to influenza a virus in human milk: a randomized trial of antenatal influenza immunization. PLoS ONE.

[15] Perl S.H., Uzan-Yulzari A., Klainer H., Asiskovich L., Youngster M., Rinott E., Youngster I. (2021). SARS-CoV-2-specific antibodies in breast milk after COVID-19 vaccination of breastfeeding women. JAMA.

[16] Gray K.J., Bordt E.A., Atyeo C., Deriso E., Akinwunmi B., Young N., Baez A.M., Shook L.L., Cvrk D., James K. (2021). Coronavirus disease 2019 vaccine response in pregnant and lactating women: a cohort study. Am. J. Obstet. Gynecol..

[17] Baird J.K., Jensen S.M., Urba W.J., Fox B.A., Baird J.R. (2021). SARS-CoV-2 antibodies detected in mother’s milk post-vaccination. J. Hum. Lact..

[18] Golan Y., Prahl M., Cassidy A.G., Gay C., Wu A.H.B., Jigmeddagva U. (2021). COVID-19 mRNA vaccination in lactation: assessment of adverse events and vaccine related antibodies in mother-infant dyads. Front. Immunol..

[19] Atyeo C., DeRiso E.A., Davis C., Bordt E.A., De Guzman R.M., Shook L.L., Yonker L.M., Fasano A., Akinwunmi B., Lauffenburger D.A. (2021). COVID-19 mRNA vaccines drive differential antibody Fc-functional profiles in pregnant, lactating, and nonpregnant women. Sci. Transl. Med..

[20] Bertotto A., Gerli R., Fabietti G., Crupi S., Arcangeli C., Scalise F., Vaccaro R. (1990). Human breast milk T lymphocytes display the phenotype and functional characteristics of memory T cells. Eur. J. Immunol..

[21] Sabbaj S., Ghosh M.K., Edwards B.H., Leeth R., Decker W.D., Goepfert P.A., Aldrovandi G.M. (2005). Breast milk-derived antigen-specific CD8+ T cells: an extralymphoid effector memory cell population in humans. J. Immunol..

[22] Tuaillon E., Valea D., Becquart P., Al Tabaa Y., Meda N., Bollore K., Van de Perre P., Vendrell J.P. (2009). Human milk-derived B cells: a highly activated switched memory cell population primed to secrete antibodies. J. Immunol..

[23] Valea D., Tuaillon E., Al Tabaa Y., Rouet F., Rubbo P.A., Meda N., Foulongne V., Bollore K., Nagot N., Van de Perre P., Vendrell J.P. (2011). CD4+ T cells spontaneously producing human immunodeficiency virus type I in breast milk from women with or without antiretroviral drugs. Retrovirology.

[24] Wirt D.P., Adkins L.T., Palkowetz K.H., Schmalstieg F.C., Goldman A.S. (1992). Activated and memory T lymphocytes in human milk. Cytometry.

[25] Morzel M., Palicki O., Chabanet C., Lucchi G., Ducoroy P., Chambon C., Nicklaus S. (2011). Saliva electrophoretic protein profiles in infants: changes with age and impact of teeth eruption and diet transition. Arch. Oral Biol..

[26] Al-Shehri S.S., Knox C.L., Liley H.G., Cowley D.M., Wright J.R., Henman M.G., Hewavitharana A.K., Charles B.G., Shaw P.N., Sweeney E.L., Duley J.A. (2015). Breastmilk-saliva interactions boost innate immunity by regulating the oral microbiome in early infancy. PLoS ONE.

[27] Karhumaa P., Leinonen J., Parkkila S., Kaunisto K., Tapanainen J., Rajaniemi H. (2001). The identification of secreted carbonic anhydrase VI as a constitutive glycoprotein of human and rat milk. Proc. Natl. Acad. Sci. U S A.

[28] Saleem B., Okogbule-Wonodi A.C., Fasano A., Magder L.S., Ravel J., Kapoor S., Viscardi R.M. (2017). Intestinal barrier maturation in very low birthweight infants: relationship to feeding and antibiotic exposure. J. Pediatr..

[29] Molès J.P., Tuaillon E., Kankasa C., Bedin A.S., Nagot N., Marchant A., McDermid J.M., Van de Perre P. (2018). Breastmilk cell trafficking induces microchimerism-mediated immune system maturation in the infant. Pediatr. Allergy Immunol..

[30] Kinder J.M., Jiang T.T., Ertelt J.M., Xin L., Strong B.S., Shaaban A.F., Way S.S. (2015). Cross-generational reproductive fitness enforced by microchimeric maternal cells. Cell.

[31] Stikvoort A., Sundin M., Uzunel M., Gertow J., Sundberg B., Schaffer M., Mattsson J., Uhlin M. (2016). Long-term stable mixed chimerism after hematopoietic stem cell transplantation in patients with non-malignant disease, shall we be tolerant?. PLoS ONE.

[32] Kalimuddin S., Tham C.Y.L., Qui M., de Alwis R., Sim J.X.Y., Lim J.M.E., Tan H.C., Syenina A., Zhang S.L., Le Bert N. (2021). Early T cell and binding antibody responses are associated with COVID-19 RNA vaccine efficacy onset. Med (N Y).

[33] Skelly D.T., Harding A.C., Gilbert-Jaramillo J., Knight M.L., Longet S., Brown A., Adele S., Adland E., Brown H., Medawar Laboratory Team (2021). https://www.researchsquare.com/article/rs-226857/v1.

[34] Collier A.Y., McMahan K., Yu J., Tostanoski L.H., Aguayo R., Ansel J., Chandrashekar A., Patel S., Apraku Bondzie E., Sellers D. (2021). Immunogenicity of COVID-19 mRNA vaccines in pregnant and lactating women. JAMA.

[35] Polack F.P., Thomas S.J., Kitchin N., Absalon J., Gurtman A., Lockhart S., Perez J.L., Pérez Marc G., Moreira E.D., Zerbini C., C4591001 Clinical Trial Group (2020). Safety and efficacy of the BNT162b2 mRNA COVID-19 vaccine. N. Engl. J. Med..

[36] Amit S., Regev-Yochay G., Afek A., Kreiss Y., Leshem E. (2021). Early rate reductions of SARS-CoV-2 infection and COVID-19 in BNT162b2 vaccine recipients. Lancet.

[37] Gonçalves J., Sousa R.L., Jacinto M.J., Silva D.A., Paula F., Sousa R., Zahedi S., Carvalho J., Cabral M.G., Costa M. (2020). Evaluating SARS-CoV-2 seroconversion following relieve of confinement measures. Front. Med. (Lausanne).

[38] Goel R.R., Apostolidis S.A., Painter M.M., Mathew D., Pattekar A., Kuthuru O., Gouma S., Hicks P., Meng W., Rosenfeld A.M. (2021). Distinct antibody and memory B cell responses in SARS-CoV-2 naïve and recovered individuals following mRNA vaccination. Sci. Immunol..

[39] Viana J.F., Bergman M., Gonçalves L.A., Duarte N., Coutinho T.P., Borges P.C., Diwo C., Castro R., Matoso P., Malheiro V. (2021). Population homogeneity for the antibody response to COVID- 19 BNT162b2 / Comirnaty vaccine is only reached after the second dose, across all adult age ranges. medRxiv.

[40] Baden L.R., El Sahly H.M., Essink B., Kotloff K., Frey S., Novak R., Diemert D., Spector S.A., Rouphael N., Creech C.B., COVE Study Group (2021). Efficacy and safety of the mRNA-1273 SARS-CoV-2 vaccine. N. Engl. J. Med..

[41] Wilson E., Butcher E.C. (2004). CCL28 controls immunoglobulin (Ig)A plasma cell accumulation in the lactating mammary gland and IgA antibody transfer to the neonate. J. Exp. Med..

[42] Tang M.W., Garcia S., Gerlag D.M., Tak P.P., Reedquist K.A. (2017). Insight into the endocrine system and the immune system: a review of the inflammatory role of prolactin in rheumatoid arthritis and psoriatic arthritis. Front. Immunol..

[43] Altmann D.M., Boyton R.J. (2020). SARS-CoV-2 T cell immunity: specificity, function, durability, and role in protection. Sci. Immunol..

[44] Sariol A., Perlman S. (2020). Lessons for COVID-19 immunity from other coronavirus infections. Immunity.

[45] Dan J.M., Mateus J., Kato Y., Hastie K.M., Yu E.D., Faliti C.E., Grifoni A., Ramirez S.I., Haupt S., Frazier A. (2021). Immunological memory to SARS-CoV-2 assessed for up to 8 months after infection. Science.

[46] Grifoni A., Weiskopf D., Ramirez S.I., Mateus J., Dan J.M., Moderbacher C.R., Rawlings S.A., Sutherland A., Premkumar L., Jadi R.S. (2020). Targets of T cell responses to SARS-CoV-2 coronavirus in humans with COVID-19 disease and unexposed individuals. Cell.

[47] Mateus J., Grifoni A., Tarke A., Sidney J., Ramirez S.I., Dan J.M., Burger Z.C., Rawlings S.A., Smith D.M., Phillips E. (2020). Selective and cross-reactive SARS-CoV-2 T cell epitopes in unexposed humans. Science.

[48] Liguoro I., Pilotto C., Bonanni M., Ferrari M.E., Pusiol A., Nocerino A., Vidal E., Cogo P. (2020). SARS-CoV-2 infection in children and newborns: a systematic review. Eur. J. Pediatr..

[49] Merewood A., Bode L., Davanzo R., Perez-Escamilla R. (2021). Breastfeed or be vaccinated-an unreasonable default recommendation. Lancet.

[50] Fox A., Marino J., Amanat F., Krammer F., Hahn-Holbrook J., Zolla-Pazner S., Powell R.L. (2020). Robust and specific secretory IgA against SARS-CoV-2 detected in human milk. iScience.

[51] Sheikh-Mohamed S., Isho B., Chao G.Y.C., Zuo M., Nahass G.R., Salomon-Shulman R.E., Blacker G., Fazel-Zarandi M., Rathod B., Colwill K. (2021). A mucosal antibody response is induced by intra-muscular SARS-CoV-2 mRNA vaccination. medRxiv.

[52] Pace R.M., Williams J.E., Järvinen K.M., Belfort M.B., Pace C.D.W., Lackey K.A., Gogel A.C., Nguyen-Contant P., Kanagaiah P., Fitzgerald T. (2021). Characterization of SARS-CoV-2 RNA, antibodies, and neutralizing capacity in milk produced by women with COVID-19. MBio.

[53] Woof J.M., Mestecky J. (2005). Mucosal immunoglobulins. Immunol. Rev..

[54] Sterlin D., Mathian A., Miyara M., Mohr A., Anna F., Claër L., Quentric P., Fadlallah J., Devilliers H., Ghillani P. (2021). IgA dominates the early neutralizing antibody response to SARS-CoV-2. Sci. Transl. Med..

[55] Wang Z., Lorenzi J.C.C., Muecksch F., Finkin S., Viant C., Gaebler C., Cipolla M., Hoffmann H.H., Oliveira T.Y., Oren D.A. (2021). Enhanced SARS-CoV-2 neutralization by dimeric IgA. Sci. Transl. Med..

[56] Miller R.A. (1941). Observations on the gastric acidity during the first month of life. Arch. Dis. Child..

[57] Wills L., Paterson D. (1926). A study of gastric acidity in infants. Arch. Dis. Child..

[58] Rydyznski Moderbacher C., Ramirez S.I., Dan J.M., Grifoni A., Hastie K.M., Weiskopf D., Belanger S., Abbott R.K., Kim C., Choi J. (2020). Antigen-specific adaptive immunity to SARS-CoV-2 in acute COVID-19 and associations with age and disease severity. Cell.

[59] Tan A.T., Linster M., Tan C.W., Le Bert N., Chia W.N., Kunasegaran K., Zhuang Y., Tham C.Y.L., Chia A., Smith G.J.D. (2021). Early induction of functional SARS-CoV-2-specific T cells associates with rapid viral clearance and mild disease in COVID-19 patients. Cell Rep..

[60] McMahan K., Yu J., Mercado N.B., Loos C., Tostanoski L.H., Chandrashekar A., Liu J., Peter L., Atyeo C., Zhu A. (2021). Correlates of protection against SARS-CoV-2 in rhesus macaques. Nature.

[61] Song G.G., Lee Y.H. (2017). Circulating prolactin level in systemic lupus erythematosus and its correlation with disease activity: a meta-analysis. Lupus.

[62] Tang M.W., Garcia S., Malvar Fernandez B., Gerlag D.M., Tak P.P., Reedquist K.A. (2017). Rheumatoid arthritis and psoriatic arthritis synovial fluids stimulate prolactin production by macrophages. J. Leukoc. Biol..

[63] Tomio A., Schust D.J., Kawana K., Yasugi T., Kawana Y., Mahalingaiah S., Fujii T., Taketani Y. (2008). Prolactin can modulate CD4+ T-cell response through receptor-mediated alterations in the expression of T-bet. Immunol. Cell Biol..

[64] Mackern-Oberti J.P., Jara E.L., Riedel C.A., Kalergis A.M. (2017). Hormonal modulation of dendritic cells differentiation, maturation and function: implications for the initiation and progress of systemic autoimmunity. Arch. Immunol. Ther. Exp. (Warsz.).

[65] Saha S., Gonzalez J., Rosenfeld G., Keiser H., Peeva E. (2009). Prolactin alters the mechanisms of B cell tolerance induction. Arthritis Rheum..

[66] Hilfiker-Kleiner D., Kaminski K., Podewski E., Bonda T., Schaefer A., Sliwa K., Forster O., Quint A., Landmesser U., Doerries C. (2007). A cathepsin D-cleaved 16 kDa form of prolactin mediates postpartum cardiomyopathy. Cell.

[67] Charepe N., Gonçalves J., Juliano A.M., Lopes D.G., Canhão H., Soares H., Serrano E.F. (2021). COVID-19 mRNA vaccine and antibody response in lactating women: a prospective cohort study. BMC Pregnancy Childbirth.

[68] Amaral-Silva D., Gonçalves R., Torrão R.C., Torres R., Falcão S., Gonçalves M.J., Araújo M.P., Martins M.J., Lopes C., Neto A. (2021). Direct tissue-sensing reprograms TLR4^+^ Tfh-like cells inflammatory profile in the joints of rheumatoid arthritis patients. Commun. Biol..

[69] Stadlbauer D., Amanat F., Chromikova V., Jiang K., Strohmeier S., Arunkumar G.A., Tan J., Bhavsar D., Capuano C., Kirkpatrick E. (2020). SARS-CoV-2 seroconversion in humans: a detailed protocol for a serological assay, antigen production, and test setup. Curr. Protoc. Microbiol..

[70] Stadlbauer D., Tan J., Jiang K., Hernandez M.M., Fabre S., Amanat F., Teo C., Arunkumar G.A., McMahon M., Capuano C. (2021). Repeated cross-sectional sero-monitoring of SARS-CoV-2 in New York City. Nature.

[71] Alenquer M., Ferreira F., Lousa D., Valério M., Medina-Lopes M., Bergman M.L., Gonçalves J., Demengeot J., Leite R.B., Lilue J. (2021). Signatures in SARS-CoV-2 spike protein conferring escape to neutralizing antibodies. PLoS Pathog..

