# Supplementary material for: Secretory IgA and T cells targeting SARS-CoV-2 spike protein are transferred to the breastmilk upon mRNA vaccination
Source: Cell Rep Med. 2021 Dec 2;2(12):100468. doi: 10.1016/j.xcrm.2021.100468 (PMC8636305; doi:10.1016/j.xcrm.2021.100468)
Supplement: Document S1. Figure S1 and Tables S1–S3 [file mmc1.pdf]

Cell Reports Medicine, Volume *2*

Supplemental information

Secretory IgA and T cells targeting SARS-CoV-2 spike protein are transferred to the breastmilk upon mRNA vaccination

Juliana Gonçalves, A. Margarida Juliano, Nádia Charepe, Marta Alenquer, Diogo Athayde, Filipe Ferreira, Margarida Archer, Maria João Amorim, Fátima Serrano, and Helena Soares

Figure S1

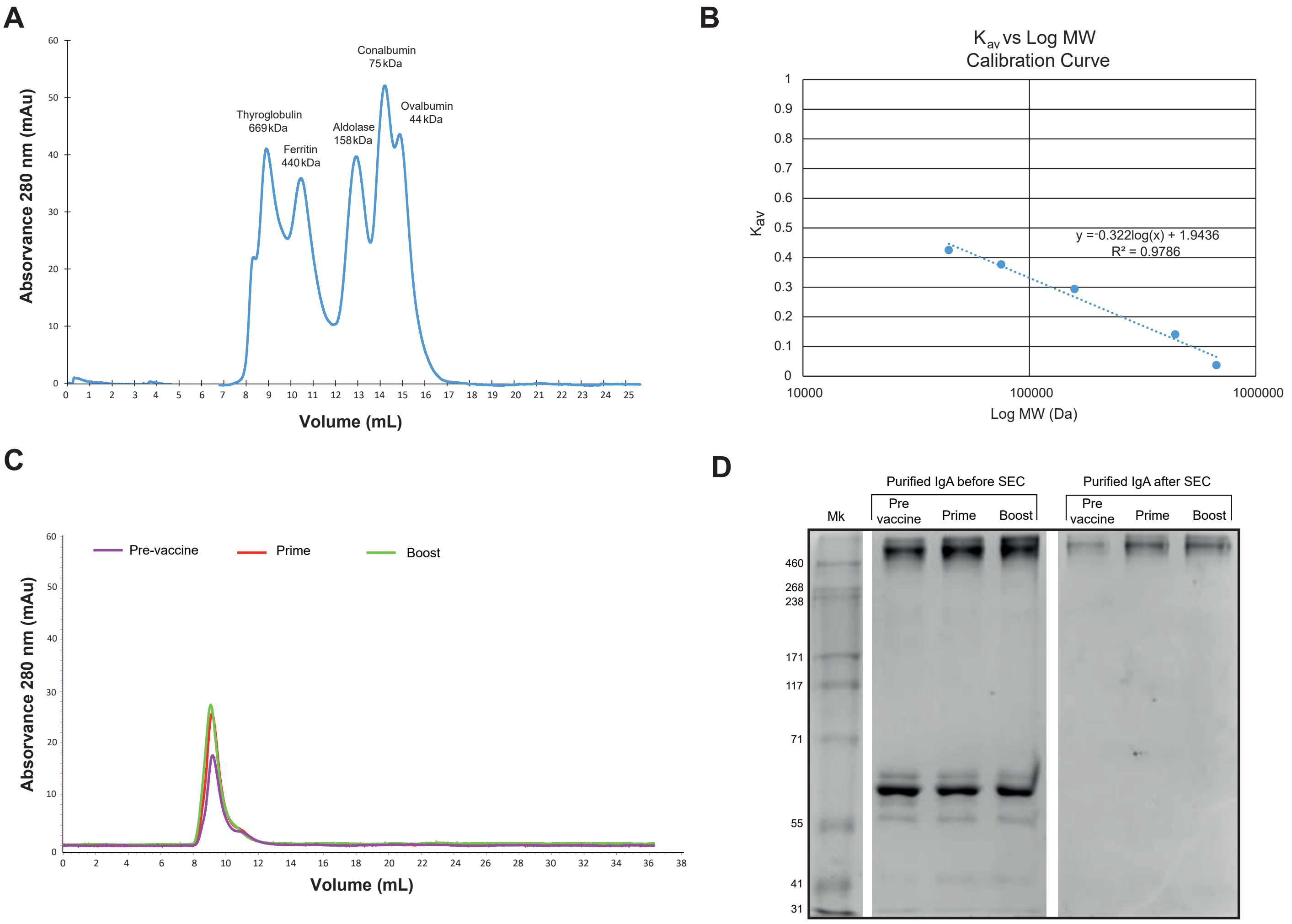

Figure S1. **Size exclusion chromatography (SEC) of IgA purified milk fractions.** Related to Figure 2. (A) Chromatogram of all standard proteins run in a Superdex 200 increase 10/300 GL. HWM Filtration Calibration Kit (Cytiva) were used with the following proteins: thyroglobulin (669 kDa); ferritin (450 kDa), aldolase (158 kDa), conalbumin (75 kDa) and ovalbumin (44 kDa). These standard proteins were dissolved in bi-distilled water and their chromatographic profiles were obtained using an UV detector. (B) Graphical representation of calibration curve of partition coefficient (Kav) of each protein versus their respective molecular weight in Daltons. The Kav was calculated through the following formula: Kav=(Ve–V0)/(Vc-V0) where Ve is the elution volume of the protein, V0 is the void volume and Vc is the column bed volume. A dispersion graph Kav vs logMW was constructed. The equation obtained for the calibration curve is: Kav = -0.322log(MW) + 1.9436, where Kav is the partition coefficient and MW is the protein molecular weight (Da). (C) Size exclusion chromatogram of skim milk purified IgA fraction collected pre-vaccine (purple), or after first (red) and second (green) vaccine doses. (D) Non-reducing polyacrylamide gel electrophoresis of purified IgA fractions before SEC and after SEC of milk samples collected pre-vaccine, or after first (prime) and second (boost) vaccine doses. For the sake of clarity we removed gel lines 2 and 5 which pertained to the paired blood sample.

Table S1. Demographic data of nursing women. Related to Figure 1.

Nursing	Age (years)	Feeding duration (months)	Days post 1st dose	Days post 2nd dose	Vaccine	COVID-19 diagnostic
1	31	16	10	10	BNT162b2	No
2	44	12	10	11	BNT162b2	No
3	34	7	8	7	BNT162b2	No
4	37	13	13	13	BNT162b2	No
5	39	21	13	10	BNT162b2	No
6	31	23	8	10	BNT162b2	No
7	33	13	8	8	BNT162b2	No
8	35	11	16	9	BNT162b2	No
9	38	4	10	10	BNT162b2	No
10	28	15	12	12	BNT162b2	No
11	33	9	9	7	BNT162b2	No
12	38	13	8	9	BNT162b2	No
13	31	3	8	9	BNT162b2	No
14	29	13	7	10	BNT162b2	No
15	30	6	12	12	BNT162b2	No
16	32	5	15	26	BNT162b2	No
17	32	4	11	21	BNT162b2	No
18	40	4	10	9	BNT162b2	No
19	30	4	15	15	mRNA-1273	No
20	31	4	11	25	BNT162b2	No
21	26	6	10	10	mRNA-1273	No
22	29	14	11	11	BNT162b2	No
23	23	9	10	10	BNT162b2	No
24*	30					
25*	28					
26*	27					

*Only pre-vaccination samples were provided and used to calculate milk Ig cut-offs.

Table S2. Demographic data of controls. Related to Figure 1.

Controls	Age (years)	Days post 2nd dose	Vaccine	COVID-19 diagnostic
1	26	10	BNT162b2	No
2	34	16	BNT162b2	No
3	26	10	BNT162b2	No
4	28	10	BNT162b2	No
5	40	10	BNT162b2	No
6	31	10	BNT162b2	No
7	30	10	BNT162b2	No
8	34	11	BNT162b2	No
9	36	11	BNT162b2	No
10*	62	11	BNT162b2	No
11	31	16	BNT162b2	No
12	31	18	BNT162b2	No
13	32	18	BNT162b2	No
14	31	21	BNT162b2	No
15	37	20	BNT162b2	No
16	39	11	BNT162b2	No
17	36	13	BNT162b2	No
18	43	9	BNT162b2	No
19	40	12	BNT162b2	No
20	23	21	mRNA-1273	No
21	25	12	mRNA-1273	No
22	43	22	BNT162b2	No
23	41	12	BNT162b2	No

*Excluded from the study due to post-menopausal status.

Table S3. OD_{450} for spike-reactive SIgA, IgA, IgG in breastmilk, and NT50 for purified milk IgA, after vaccine second dose. Related to Figure 2.

Nursing	OD_{450} anti-Spike SIgA	OD_{450} anti-Spike IgA	OD_{450} anti-Spike IgG	NT50 purified IgA
1	0.491	1.39	1.156	n.d.
2	0.249	0.899	0.988	n.d.
3	0.097	0.806	0.923	n.d.
4	0.216	1.092	0.992	n.d.
5	0.353	1.427	0.961	n.d.
6	0.159	0.853	1.088	n.d.
7	0.226	1.174	1.042	n.d.
8*	1.052	1.6	1.032	-
9	0.424	1.476	0.957	7.45 (4.29-9.57)
10	0.085	0.726	0.973	n.d.
11	0.269	1.258	1.174	n.d.
12	0.204	1.099	1.057	n.d.
13	0.137	0.746	0.963	n.d.
14	0.266	0.975	1.043	n.d.
15	0.102	0.145	0.86	n.d.
16	0.062	0.087	0.763	n.d.
17	0.205	0.313	1.037	n.d.
18	0.167	0.796	0.869	n.d.
19	0.834	1.339	0.945	2.15 (0.14-6.47)
20	0.301	0.831	0.484	n.d.
21	0.665	1.359	0.881	9.15 (4.22-39.97)
22	0.109	1.012	0.615	n.d.
23	0.133	0.627	1.191	n.d.

*This sample could not be used to purify milk IgA due to insufficient volume.
